# Treating cancer with amplitude-modulated electromagnetic fields: a potential paradigm shift, again?

**DOI:** 10.1038/bjc.2011.576

**Published:** 2012-01-17

**Authors:** C F Blackman

**Affiliations:** 1 Integrated Systems Toxicology Division (B-105-03), US Environmental Protection Agency, Research Triangle Park, NC 27711, USA

The Zimmerman *et al* (2012) study published here, coupled with the group's two preceding papers (Barbault *et al*, 2009; Costa *et al*, 2011), identify a potential modality for treating tumours at a dramatic reduction in trauma and cost. This set of clinical and explanatory laboratory results should be understood in the context of the history of research into the biological effects of electromagnetic fields (EMFs).

The most successful clinical application is the use of EMF to initiate fusion in fractured long bones that would not otherwise heal. Pulsed fields were designed to simulate the natural piezoelectric signals generated from bones under varying stress while walking (e.g., Bassett, 1985). There are also other reports that EMF can reduce pain and stimulate wound healing after surgery.

The group's two previous clinical reports were critical to the design of this new Zimmerman *et al* study. Barbault *et al* (2009) described how they obtained the specific frequencies for different tumour diagnoses, which are then used in the amplitude-modulated (AM)-EMF treatment of those patients to stabilise the disease beyond normal expectations. Costa *et al* (2011) reported surprising clinical benefits from using the specific AM-EMF signals to treat advanced hepatocellular carcinoma, stabilising the disease and even producing partial responses up to 58 months in a subset of the patients. Now Zimmerman *et al* have examined the growth rate of human tumour cell lines from liver and breast cancers along with normal cells from those tissues exposed to AM-EMF. Reduced growth rate was observed for tumour cells exposed to tissue-specific AM-EMF, but no change in growth rate in normal cells derived from the same tissue type, or in tumour or normal cells from the other tissue type. The growth rate inhibitory response was field-strength (SAR) and exposure-time dependent. In ancillary tests, they observed reduction in gene expression and increases in mitotic spindle dysfunction only for the AM-EMF exposure that reduced the cell growth rate.

The work of Zimmerman *et al*, Costa *et al* and Barbault *et al* was not done in a vacuum. More than 30 years ago, Suzanne Bawin working in Ross Adey's lab (Bawin *et al*, 1975), with independent replication by my group (Blackman *et al*, 1979), demonstrated that biological effects could be caused by certain AM frequencies on a carrier wave but not other frequencies, similar to the current work. Subsequent reports in the 1980s by several groups continued to support and extend the initial findings (Adey, 1992; Blackman, 1992).

This growing collection of reports demonstrating AM-EMF-induced biological effects led to recognition by national and international authorities that this modality needed to be considered in hazard evaluation, in addition to field-induced heating as a cause for health concern. The National Council on Radiation Protection and Measurements (1986) recommended a reduction in the allowable exposure intensity limits for AM radiation above a certain level, and the World Health Organization (1993) explicitly acknowledged AM as a future issue to be examined in setting exposure guidelines. Unexpectedly, research funding for this area dried up around 1990 and scientific advances dramatically slowed. A promising area of research fell by the wayside.

The Zimmerman *et al* paper, providing essential laboratory data to support the two previous clinical treatment papers, has resurrected the promising AM-EMF paradigm. It should lead to a major reevaluation of this novel and potentially effective treatment for cancer and possibly other conditions. This study demonstrates the fundamental requirement for a biological ‘information content’ code (i.e., the AM spectral profile, much like different AM radio stations with different content – e.g., all news, or music) that can affect tumour cells from the tissue of origin, while apparently being ignored by normal cells from various tissues and tumour cells from different tissues of origin. The correspondence between AM-EMF-induced effects on cell proliferation, gene expression, and mitotic spindle dysfunction provide some clues to a possible biological mechanism of action.

The tools developed in Barbault *et al* (2009) to identify relevant treatment frequencies can be seen to have direct clinical and medical relevance in determining the characteristics of a new modality that may prove useful in cancer treatment. The precision of the frequency definitions, down to 1 mHz, is very unusual, but it is reminiscent of the biological effects reported for 40–48-GHz frequencies by Grundler *et al* (1982), and may represent a true effective frequency limitation that most studies would have missed, because of the lack of available, precise generation equipment or lack of the investigator knowledge.

The Zimmerman *et al* study raises a number of issues to be resolved. First, a more detailed elucidation of AM-EMF-induced genomic pathway changes is needed in order to put the results on a firmer mechanistic basis. Second, more information is needed on the nature of the growth inhibition, for example, is it persistent or do resistant cells emerge from continued treatment? Third, will cells from liver or breast tissues in different stages of transformation reduce or enhance sensitivity to AM-EMF exposure? Fourth, will tissue-specific AM-EMF tumour treatments for humans have similar effects on cells from animal tumours? For example, if rodent liver tumour cells respond similarly to the treatments, this may open a new, more rapid investigation of the therapeutic efficacy of the technique.

When the three studies are taken together, it is apparent that there are gaps in knowledge that can limit the acceptance of this treatment for cancer. How do the biofeedback endpoints (skin electrical resistance, pulse amplitude and blood pressure) engage with the disease state to provide an indication of effective frequencies to treat patients, and most surprisingly, to directly affect tumour cells *in vitro* from the same tissue type? The issue of frequency precision in the AM-EMF signal also needs to be examined and characterised as a function of different physiological growth conditions. Equally mysterious is the mechanism by which AM-EMF administered via a spoon-shaped antenna placed in the mouth can influence cancer cells in the liver or breast of patients. Finally, these patients had advanced cancer and were in palliative care when EMF testing began. Would earlier intervention in breast cancer or liver cancer cases with AM-EMF prove to be more effective?

Funding is needed for further medical and basic science research to identify and characterise the biological influence that amplitude-modulated EMFs have on the body, in its normal state, when recovering from disease or injury, and when initially affected by disease. As a caution, ‘information content’ EMF signals may not always have beneficial consequences for humans or their environment, so research should examine potential detrimental biological outcomes as well.

The group of three papers demonstrate a new, potentially important modality in the treatment of cancer that could lead to a paradigm shift in disease treatment. I hope that this medical application of AM-EMF will not be allowed languish without funding, as happened with its previous, ill-fated emergence.
